# Vitamin C Inhibits Lipopolysaccharide-Induced Hyperinflammatory State of Chronic Myeloid Leukemia Cells through Purinergic Signaling and Autophagy

**DOI:** 10.3390/nu16030383

**Published:** 2024-01-29

**Authors:** Daniela A. Pires, Maysa A. R. Brandão-Rangel, Anamei Silva-Reis, Fabiana R. S. Olímpio, Flavio Aimbire, Carlos R. Oliveira, José R. Mateus-Silva, Lucas S. Zamarioli, André L. L. Bachi, Yanesko F. Bella, Juliana M. B. Santos, Claudia Bincoletto, Antonio Herbert Lancha, Rodolfo P. Vieira

**Affiliations:** 1 Post-Graduation Program in Bioengineering, Universidade Brasil, Rua Carolina Fonseca 235, São Paulo 08230-030, SP, Brazil; danielaapires1977@hotmail.com; 2 Postgraduate Program in Science of Human Movement and Rehabilitation, Federal University of São Paulo (UNIFESP), Avenida Ana Costa 95, Santos 11060-001, SP, Brazil; maysarangel_4@hotmail.com (M.A.R.B.-R.); anameisreis97@gmail.com (A.S.-R.); yanesko@hotmail.com (Y.F.B.); juliana-mbs@hotmail.com (J.M.B.S.); 3 Department of Medicine, Postgraduate Program in Translational Medicine, Federal University of São Paulo (UNIFESP), Rua Pedro de Toledo 720, Vila Clementino, São Paulo 04039-002, SP, Brazil; fabiana.olimpio@outook.com (F.R.S.O.); flavio.aimbire@unifesp.br (F.A.); 4 Gap Biotech Laboratory of Biotechnology and Bioinformatics, Rua Comendador Remo Cesaroni 223, São José dos Campos 12243-020, SP, Brazil; carlos.rocha@unifesp.br (C.R.O.); jrm@gap.com.br (J.R.M.-S.); 5 Department of Pharmacology, Federal University of São Paulo (UNIFESP), Rua Três de Maio 100, São Paulo 04044-020, SP, Brazil; lucaszamarioli@gmail.com (L.S.Z.); claudia.bincoletto@unifesp.br (C.B.); 6 Postgraduate Program in Health Science, Santo Amaro University, Rua Prof. Enéas de Siqueira Neto 340, São Paulo 04829-300, SP, Brazil; albachi@prof.unisa.br; 7 Experimental Surgery (LIM 26), Laboratory of Clinical Investigation, School of Medicine, University of Sao Paulo, Avenida Doutor Arnaldo 455, São Paulo 05508-030, SP, Brazil; lanchajunior@gmail.com; 8 Postgraduate Program in Human Movement and Rehabilitation and in Pharmaceutical Sciences, Evangelical University of Goiás (Unievangélica), Avenida Universitária Km 3,5, Anápolis 75083-515, GO, Brazil

**Keywords:** ascorbic acid, vitamin C, purinergic signaling, leukemia, autophagy, inflammation

## Abstract

**Background**: Chronic myeloid leukemia (CML) is a myeloproliferative neoplasm characterized by the overproduction of white blood cells, leading to symptoms such as fatigue, infections, and other complications. CML patients must take measures to prevent infections to mitigate the exacerbation of cancer cell proliferation and comorbidities. **Methods**: This study investigated whether vitamin C can suppress the hyperinflammatory activation of K-562 cells induced by lipopolysaccharide (LPS) and whether purinergic signaling (ATP and P2X7 receptor) and autophagy play a role in it. Two different doses of vitamin C (5 µg/mL and 10 µg/mL) were employed, along with the lysosome inhibitor chloroquine (CQ; 100 µM), administered 2 h prior to LPS stimulation (10 ng/mL) for a duration of 22 h in K-562 cells (3 × 10^5^ cells/mL/well). **Results**: Both doses of vitamin C reduced the release of interleukin-6 (IL-6) (5 µg/mL, *p* < 0.01 and 10 µg/mL, *p* < 0.01) and tumor necrosis factor (TNF) (5 µg/mL, *p* < 0.01 and 10 µg/mL, *p* < 0.01) induced by LPS. Furthermore, in LPS + CQ-stimulated cells, vitamin C at a concentration of 10 µg/mL inhibited the expression of LC3-II (*p* < 0.05). Conversely, both doses of vitamin C led to the release of the anti-inflammatory cytokine interleukin-10 (IL-10) (5 µg/mL, *p* < 0.01 and 10 µg/mL, *p* < 0.01), while only the 10 µg/mL dose of vitamin C induced the release of Klotho (10 µg/mL, *p* < 0.01). In addition, both doses of vitamin C reduced the accumulation of ATP (5 µg/mL, *p* < 0.01 and 10 µg/mL, *p* < 0.01) and decreased the expression of the P2X7 receptor at the mRNA level. **Conclusions**: Vitamin C inhibits the hyperinflammatory state induced by LPS in K-562 cells, primarily by inhibiting the ATP accumulation, P2X7 receptor expression, and autophagy signaling.

## 1. Introduction

Chronic myeloid leukemia (CML) is a myeloproliferative neoplasm that is associated with a cytogenetic abnormality on the Philadelphia (Ph) chromosome, which results in a reciprocal translocation between chromosomes 9 and 22. CML primarily affects adults and has an incidence of 1–2 cases per 100,000 adults [1,2].

This disorder begins with a non-lethal overproduction of leukocytes, which subsequently progresses to leukemia [1,2,3]. This transformation is marked by accentuated leukocytosis with a predominance of immature myeloid precursors and sometimes platelets [1,2,3]. It is important to note that CML develops slowly and progressively. Signs and symptoms typically manifest when the leukocyte count reaches 30 to 90 thousand [1,2,3]. CML patients experience an overproduction of white blood cells, often characterized by a gradual increase in the number of transformed cells over time. This can lead to symptoms such as anemia, fatigue, infections, bleeding, and other complications [1,2,3]. However, some patients remain completely asymptomatic, and CML is frequently detected during routine hematological examinations [3].

According to the literature, a significant increase in the levels of pro-inflammatory mediators, primarily pro-inflammatory cytokines like interleukin (IL)-6 and tumor necrosis factor (TNF), is closely associated with increased proliferation and enhanced survival of cancer cells. This elevation may also indicate the hyperactivation of myeloid cancer cells, including those in CML [4]. In fact, patients with different types of cancer are much more susceptible to infections, which result in increased levels of pro-inflammatory cytokines [5,6]. Furthermore, even CML patients in remission still respond negatively in cases of infections, such as SARS-CoV-2 virus infections [7,8].

On the other hand, another noteworthy aspect of this disease is that, due to elevated neutrophil counts and the preservation of humoral and cellular immune responses, lower infection rates are observed in the chronic phase of CML [8]. However, in the initial months of treatment, CML patients experience a significant increased risk of infections due to neutropenia observed during this period [8]. Currently, the primary therapy for CML is based on tyrosine kinase inhibitors (TKIs), which strongly interfere with the interaction between the BCR-ABL1 oncoprotein and adenosine triphosphate (ATP), leading to the blockade of malignant clone proliferation [1,8]. For example, imatinib mesylate selectively inhibits the BCR-ABL1 oncoprotein through competing with ATP to bind to BCR-ABL1 [9]. In addition, another in vitro study investigating the involvement of purinergic signaling in bacterial infections in chronic myeloid leukemia (CML) using the K-562 cell line demonstrated that an herbal medicine named Immunity-6^®^, in the context of lipopolysaccharide (LPS) bacterial infection, exhibited that while LPS increased adenosine triphosphate (ATP) release and accumulation, as well as the expression of the P2X7 receptor, the treatment with Immunity-6^®^ reduced both ATP release and accumulation, along with P2X7 receptor expression [10], reinforcing the involvement of these components of purinergic signaling in CML. In fact, P2X7 receptor hyperexpression modulates cell proliferation, inflammation, and fibrosis [10].

In addition to what has been mentioned, the involvement of autophagy in CML pathophysiology and progression was reported in a study where the authors observed an increase in the autophagic response following treatment with imatinib mesylate (IM) [11]. Similar effects have been observed with other non-targeting drugs in CML cell lines and primary patient samples [12]. These effects were associated with increases in the generation of reactive oxygen species (ROS), endoplasmic reticulum (ER) stress, and a dose-dependent upregulation of key autophagy regulators in terms of both genes and proteins [11,13,14]. Furthermore, it has been demonstrated that autophagy induces cellular senescence in CML cells rather than apoptosis, rendering them more resistant to treatment [15].

Interestingly, in this context, it was reported that vitamin C shows the ability to restores cellular sensitivity to imatinib, which can amplify the capacity of this BCR/ABL tyrosine kinase inhibitor (TKI) to promote a reduction in proliferation of the malignant cells [16]. In terms of vitamin C, even though its deficiency is uncommon in the general population, patients with cancer often present reduced levels of this vitamin [17]. In this sense, it has been reported that lower cellular levels of vitamin C may facilitate the normal cells to turn into leukemia cells [18] while high doses of vitamin C may cause leukemic cells to die [16]. Furthermore, vitamin C supplementation can also putatively act to improve health-related quality of life in leukemia patients [19]. In addition, the initial evidence suggests that vitamin C down-regulates the purinergic signaling, which is the main hypothesis of the present study and was tested on the context of CML [20]. Beyond that, the literature already shows that vitamin C up-regulates autophagy [21], with increases in autophagy are believed to reduce leukemic cell viability and enhance sensitivity to standard chemotherapy [22]. So, the present study investigated whether the effects of vitamin C on CML cells may involve components of the purinergic and autophagy signaling pathways.

Considering that TKIs, like imatinib, exhibit significant crosstalk with purinergic signaling and autophagy, and acknowledging that vitamin C can act on the same signaling pathway, the present study aimed to investigate whether vitamin C could inhibit the inflammatory response of chronic myeloid cells to LPS by modulating components of the purinergic signaling and autophagy pathways. In summary, the present study demonstrated that vitamin C is capable of inhibiting LPS-induced pro-inflammatory responses in CML cells through the downregulation of components of the purinergic signaling and autophagy pathways.

## 2. Material and Methods

### 2.1. Human Cell Culture Study

The K-562 cell line, consisting of human blood (chronic myelogenous leukemia) cells, was obtained from the Rio de Janeiro Cell Bank. After growing the cells until enough cells were obtained, at the 8th passage, the cells were used for the experiments. The K-562 cells were cultured in DMEM high-glucose medium (Sigma Chemical Co., St. Louis, MO, USA), supplemented with 10% fetal calf serum with 100 μg/mL of streptomycin and 100 UI/mL of penicillin. The cells were seeded at a concentration of 3 × 10^5^/mL/well in 48-well plates and grown in a humidified atmosphere in a CO^2^ incubator (5% CO_2_, 37 °C) [23].

The cells were pre-treated with vitamin C at two different dosages (5 µg/mL and 10 µg/mL; Sigma Chemical Co., St. Louis, MO, USA) and/or with the lysosome inhibitor chloroquine (CQ; 100 µM, C6628-25G, Sigma Aldrich, St. Louis, MO, USA) for 2 h and then stimulated with lipopolysaccharide from Gram-negative bacteria (LPS; 10 ng/mL; Escherichia coli 026:B6; L3755, Sigma Aldrich, St. Louis, MO, USA) for 22 h. The cells and supernatant were recovered, centrifuged at 900× *g* for 5 min at 4 °C, and then the supernatant was immediately used for ATP measurements and later (after frozen the supernatant at −86 °C) for cytokine measurements. The cells were washed using phosphate-buffered saline (PBS) and then subjected to the RT-PCR protocol. All experiments were performed in triplicate and repeated thrice.

### 2.2. Cytotoxicity Evaluation Using MTT Assay

To assess the vitamin C cytotoxicity in our study, the cell viability was evaluated using the MTT assay. Briefly, 5 × 10^4^ viable K-562 cells were placed into clear 96-well flat-bottom plates (Corning, New York, NY, USA) in DMEM high-glucose medium supplemented with 10% fetal calf serum and immediately after, different concentrations (0.1; 1; 10; 100; and 1000 μg/mL) of vitamin C were added to the cells. Following a 24 h incubation in a humidified atmosphere in a CO^2^ incubator (5% CO_2_, 37 °C), 10 μL/well of MTT (5 mg/mL) was added to the cells (both in the control and vitamin C and CQ treatments), which were incubated for an additional 4 h. After this time, 100 μL of a 10% sodium dodecyl sulfate (SDS) solution in deionized water was added to the cells and incubated overnight [23]. The absorbance was measured at 595 nm using a benchtop multimode SpectraMax i3 reader (Molecular Devices, San Jose, CA, USA).

### 2.3. Adenosine Triphosphate (ATP) Measurement

The ATP concentration was determined in the cell culture supernatant immediately after its collection using the ATPlite Luminescence Assay System (Perkin Elmer, Waltham, MA, USA) according to the manufacturer’s instructions. The results were expressed in mmol/mL [24].

### 2.4. Cytokines Measurement

The levels of interleukin (IL)-1RA (DY280), IL-6 (DY206), IL-10 (DY217), TNF-alpha (DY210), and Klotho (DY5334-05) were measured in the cell culture supernatant using the DuoSet ELISA kit (R&D Systems; Minneapolis, MN, USA) according to the manufacturer’s recommendations. The results were expressed as pg/mL [24].

### 2.5. Reverse Transcriptase–Polymerase Chain Reaction (RT-PCR)

The total RNA of treated and untreated K-562 cells was extracted using Trizol^®^ (GIBCO-BRL, Gaithersburg, MD, USA), according to classical protocol following the manufacturer’s instructions. β-actin was used as a control and correction factor for the expression of the P2X7 receptor, for which the sequences of primers are as follows: β-actin forward (5′GTGGGCCGCTCTAGGCACCA3′) and reverse primers (5′CTCTTTGATGTCACGCACGATTTC3′ 540 bp) [25] and P2X7 forward (5′-AGATCGTGGAGAATGGAGTG-3′) and reverse primers (5′-TTCTCGTGGTGTAGTTGTGG-3′) [26].

### 2.6. Protein Expression of Autophagy Biomarker LC3-I and LC3-II

We employed Western blotting to detect the presence of LC3-I, LC3-II, and the reference protein α-tubulin. In brief, K-562 cells were aliquoted into 2 mL tubes (3 × 10^5^ cells per tube) and washed three times with PBS and subjected to centrifugation to obtain a cell pellet. The cell pellet was lysed using RIPA Lysis Buffer (Thermo Scientific, cod 89901, Waltham, MA, USA) and the proteins were quantified using the BCA method [27]. The lysate samples were then separated by electrophoresis on 10% SDS-PAGE gels and subsequently transferred onto nitrocellulose membranes (Bio-Rad, Hercules, CA, USA). The membranes were blocked for 1 h at room temperature using a solution of 5% non-fat milk and 0.05% Tween-20 in phosphate-buffered saline. The membranes were then incubated overnight at 4 °C with primary antibodies against LC3B (dilution 1:500; code #2775; Cell Signaling, Danvers, MA, USA) or α-tubulin (1:500; code #T8203; Sigma Aldrich, Saint Louis, MO, USA). After incubation, the membranes were washed and exposed to horseradish peroxidase-conjugated secondary antibodies at room temperature for 2 h. Immunoreactive bands were visualized using a chemiluminescence detection system (Imuno-Star, Bio-Rad, CA, USA). The relative band intensities were quantified using ImageJ software (Accessed on 12 July 2023 on https://imagej.net/ij/download.html, National Institutes of Health, Bethesda, MD, USA) and normalized to the intensity of the α-tubulin band. The experiments were performed in triplicate and repeated thrice. For electrophoresis, three samples were pooled, resulting in three final samples per group.

### 2.7. Statistical Analysis

We conducted statistical analysis and created graphs using GraphPad Prism 5.0 software. The results are presented as mean values with the standard error of the mean (SEM) based on data from three independent experiments. Multiple comparisons were performed using one-way analysis of variance (ANOVA), followed by the Bonferroni post hoc test to assess group differences. A *p*-value of less than 0.05 was considered statistically significant.

## 3. Results

### 3.1. High Levels of Vitamin C Induced K-562 Cell Toxicity

**Figure 1:**
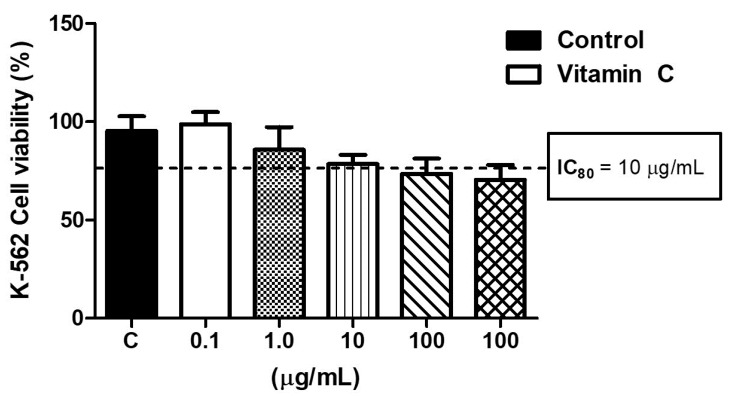
Effects of different doses of vitamin C (0.1, 1, 10, 100, 1000 µM) on cell viability to determine the dose able to reduce the cell viability by 20% (IC80). All experiments were performed in triplicate and repeated thrice.

Figure 1 shows the effects of different dosages of vitamin C (0.1, 1, 10, 100, and 1000 µg/mL) on K-562 cell viability. Considering the IC 80, which represents the dosage required to reduce the cell viability by 20%, it was found that only the higher vitamin C dosages (>10 µg/mL) induced the same viability level as the IC 80.

### 3.2. Vitamin C Modulates Pro- and Anti-Inflammatory Cytokines in K-562 Cells

**Figure 2:**
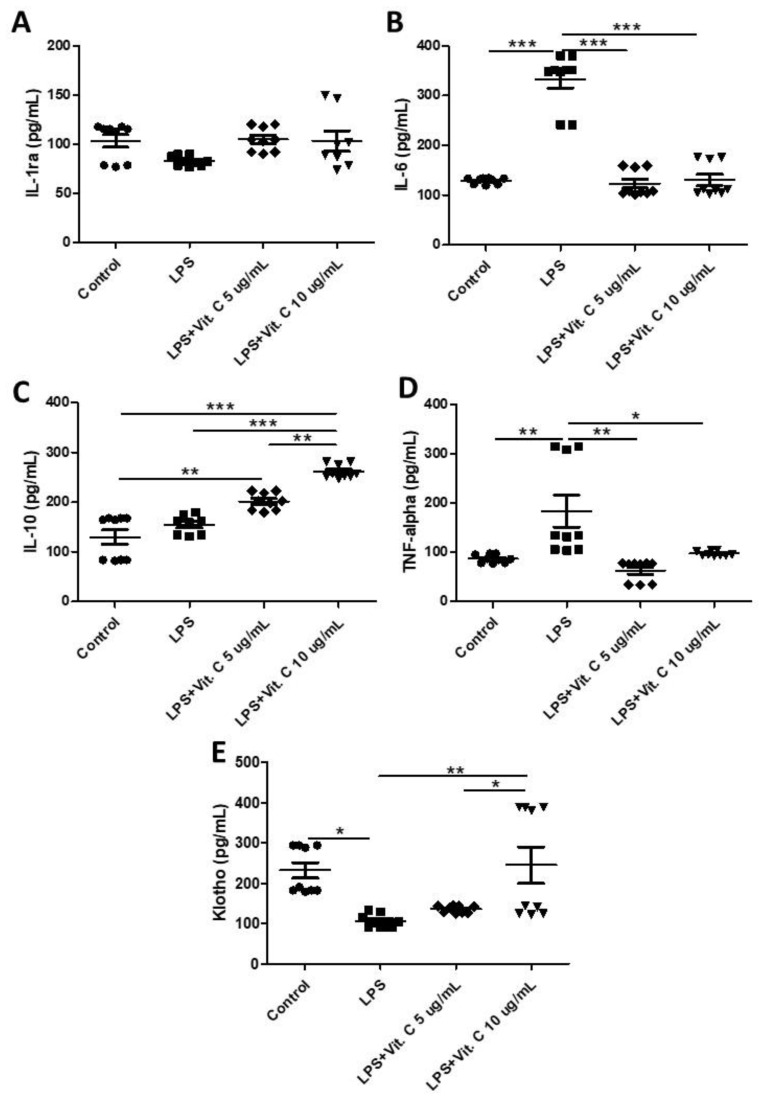
Results of the concentrations (pg/mL) of interleukin-1 receptor antagonist (IL-1ra) (**A**), IL-6 (**B**), IL-10 (**C**), tumor necrosis factor (TNF) (**D**), and Klotho (**E**) obtained in the control, and LPS-stimulated (1 µg/mL for 24 h) cells with and without pre-treatment with vitamin C (5 µg/mL or 10 µg/mL) for 2 h. All experiments were performed in triplicate and repeated thrice. Statistical analyses using two-way repeated-measures ANOVA tests are presented as the mean and standard deviation (SD). * *p* < 0.05; ** *p* < 0.01; *** *p* < 0.001.

Figure 2 shows the effects of two different dosages of vitamin C (5 µg/mL and 10 µg/mL) on the pro-inflammatory (Figure 2B, IL-6, and Figure 2D, TNF) and anti-inflammatory cytokines (Figure 2B, IL-1ra, Figure 2C, IL-10, and Figure 2E) released by K-562 cells stimulated by LPS. Among the pro-inflammatory cytokines, higher levels of IL-6 (Figure 2B, *p* < 0.01) and TNF (Figure 2D, *p* < 0.01) were found in the LPS-stimulated cell culture compared to the control, whereas the pre-treatment with vitamin C at both doses (5 µg/mL and 10 µg/mL) significantly reduced the levels of IL-6 (Figure 2B, *p* < 0.01) and TNF (Figure 2D, *p* < 0.01) in the cell cultures stimulated with LPS. Regarding the anti-inflammatory cytokines, although no differences were observed in the IL-1ra levels (Figure 1), with both LPS stimulation and vitamin C treatment, higher IL-10 levels were found in the LPS-stimulated cell culture pre-treated with 5 µg/mL of vitamin C compared to the control (Figure 2C; *p* < 0.01), as well as those pre-treated with 10 µg/mL vitamin C compared to the other cell cultures (control, LPS only, and LPS + 5 µg/mL vitamin C, Figure 2C; *p* < 0.01). In particular, for Klotho, an anti-aging and anti-inflammatory cytokine, LPS stimulation significantly inhibited its release compared to the control group (Figure 2E; *p* < 0.05); however, the pre-treatment with 10 µg/mL of vitamin C was able to prevent this inhibition with an increase in Klotho release in comparison to the values observed in the only LPS-stimulated and with LPS + 5 µg/mL vitamin C cell cultures (Figure 2E; *p* < 0.01, *p* < 0.05, respectively).

### 3.3. Vitamin C Modulates Components of the Purinergic Signaling Pathway in K-562 Cells

**Figure 3:**
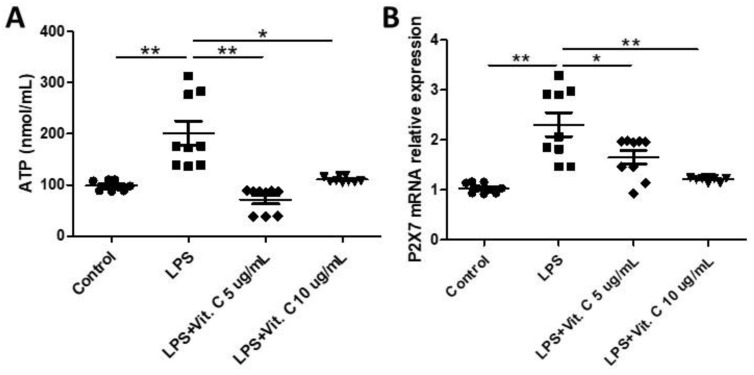
Effects of LPS stimulation and vitamin C supplementation (5 µg/mL or 10 µg/mL) on adenosine triphosphate (ATP) concentrations (**A**) in the K-562 cells pre-treated with vitamin C (5 µg/mL or 10 µg/mL) for 2 h and stimulated with LPS (10 ng/mL) for 24 h. Effects of LPS stimulation and vitamin C supplementation (5 µg/mL or 10 µg/mL) on the expression of mRNA of P2X7 receptor (**B**) in K-562 cells. All assays were performed in triplicate and repeated thrice and the mean ± standard deviations are shown. * *p* < 0.05 versus LPS-treated cells or * *p* < 0.05 versus LPS + vitamin C-treated cells. The mRNA levels were determined using real-time qRT-PCR. * *p* < 0.05; ** *p* < 0.01.

Figure 3 shows the levels of adenosine triphosphate (ATP) (Figure 3A) in the supernatant, as well as the mRNA expression of the P2X7 receptor (Figure 3B) in the K-562 cell cultures pretreated with vitamin C (5 µg/mL or 10 µg/mL) and then stimulated with LPS. Higher levels of ATP (Figure 3A) were found in the LPS-stimulated cell cultures than t in the control (*p* < 0.01), whereas a significant reduction in their levels was found in the cell cultures that were also stimulated with LPS but pretreated with vitamin C at 5 µg/mL (*p* < 0.01) and 10 µg/mL (*p* < 0.05) in comparison to the values observed in the LPS-stimulated cell culture. In the same way, LPS-stimulated cell cultures presented a higher expression of P2X7 receptors than the control (*p* < 0.01), while the cell culture pretreated with vitamin C (only for the dosage of 10 µg/mL; *p* < 0.05) showed a significant decrease in expression of the P2X7 receptor compared to the values observed in the LPS-stimulated cell culture.

### 3.4. Vitamin C Reduces Autophagy Pathway in K-562 Cells

**Figure 4:**
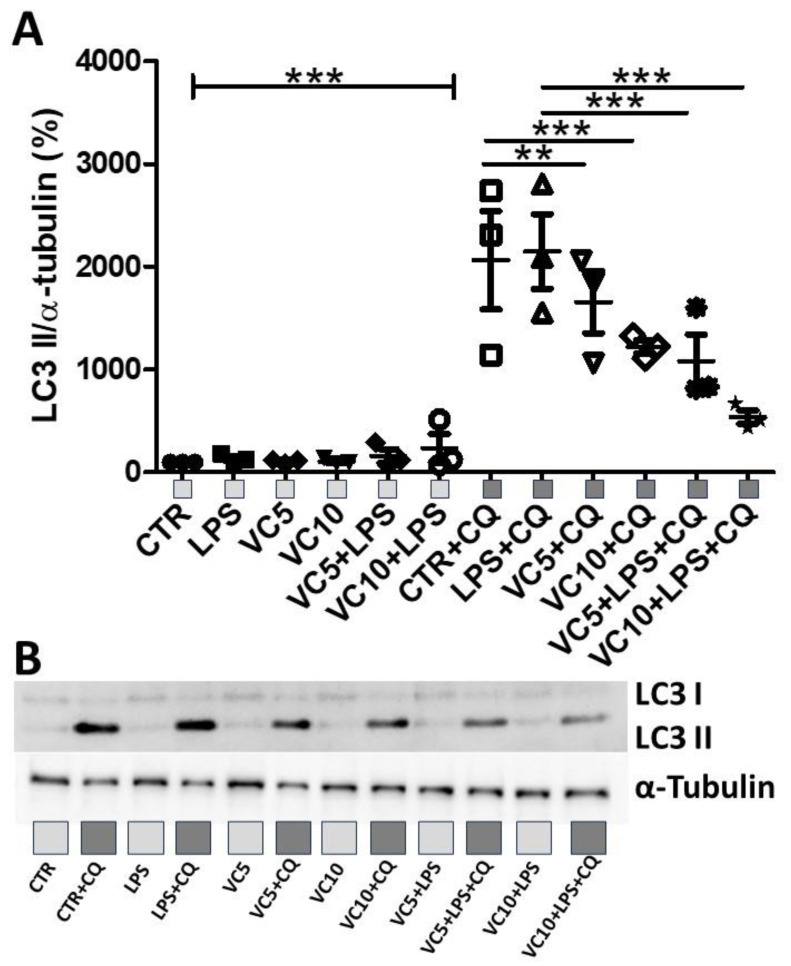
Effects of LPS stimulation and vitamin C supplementation (5 µg/mL or 10 µg/mL) on LC3-I and LC3-II expression. (**A**) Quantitative analysis of the expression of LC3-I and LC3-II normalized to α-tubulin. (**B**) Representative Western blot of the expression of LC3-I, LC3-II, and α-tubulin. CTR = control. CQ = lysosome inhibitor chloroquine. LPS = lipopolysaccharide. VC5 = vitamin C 5 µg/mL. VC10 = vitamin C 10 µg/mL. All experiments were performed in triplicate and repeated thrice. ** = *p* < 0.01; *** = *p* < 0.001. *** = *p* < 0.001 comparing all groups without CQ with all groups with CQ. Light gray square represent non-stimulated groups with CQ. Dark gray squares represent groups stimulated with CQ.

Figure 4 shows the expression of LC3-I and LC3-II in the K-562 cell cultures pretreated with vitamin C (5 µg/mL or 10 µg/mL) and/or chloroquine (CQ) and stimulated with LPS. Figure 4A shows that higher expression of LC3-II was observed in Control + Chloroquine and LPS + Chloroquine in comparison to all other groups (**** *p* < 0.0001). In addition, both doses of vitamin C in combination with chloroquine (VC5 CQ and VC10 CQ) still resulted in increased expression of LC3-II (VC5 CQ, *p* < 0.001; VC10 CQ, *p* < 0.01). Lastly, only the lower dosage of vitamin C (5 µg/mL, VC5) in LPS plus chloroquine-stimulated cells still resulted in increased expression of LC3-II compared to non-stimulated groups with chloroquine (VC5 LPS CQ, *p* < 0.05). Figure 4B shows a representative image of the Western blot.

## 4. Discussion

In this study, we successfully demonstrated, for the first time, that vitamin C treatment can prevent the hyperinflammatory activation of chronic myeloid cancer cells (K-562 cells) triggered by LPS. This was evidenced by a reduction in pro-inflammatory cytokines such as IL-6 and TNF, an increase in anti-inflammatory cytokines IL-10 and Klotho, and the inhibition of ATP accumulation, P2X7 receptor expression, and the autophagy pathway.

As previously reported, it is widely accepted that cytokines can directly influence tumor progression [4,28]. Moreover, elevated levels of pro-inflammatory cytokines, which are generally considered a hallmark of myeloid cancer cell hyperactivation, can induce not only cell proliferation but also resistance to the apoptosis process in these cancer cells [28,29].

In this context, higher levels of IL-6, a pleiotropic cytokine involved in both inflammatory responses and cancer development, have been observed in patients with various types of myeloid cancer [28,29]. Furthermore, elevated IL-6 levels can have a detrimental impact on the immune response, thereby creating a favorable microenvironment for the development of infectious processes, a situation that poses significant risks and is frequently encountered in cancer patients [29,30]. Specifically, in the case of myelomas, considering the information mentioned previously, the increased expression of the IL-6 receptor in response to heightened IL-6 release is suggested as a promising target for diagnoses and therapeutic interventions [30]. In line with the remarkable ability of myeloid cancer cells to produce pro-inflammatory cytokines when hyperactivated, our present study demonstrated that K-562 cells, when stimulated with LPS, exhibit an increased release of IL-6. Notably, as a novel finding in this study, we also established that the pretreatment of K-562 cells with vitamin C (5 µg/mL and 10 µg/mL) significantly reduced IL-6 release following LPS stimulation. These results hold significant relevance since elevated levels of IL-6 have been identified as the primary biomarker associated with pulmonary bacterial infections in chronic myeloid leukemia (CML) patients [31]. Indeed, heightened concentrations of IL-6 are commonly observed in cases of infection, and the evaluation of IL-6 levels in the bloodstream is effective in detecting the presence of an infection [32]. In individuals with sepsis, IL-6 concentrations may increase significantly, reaching up to 1600 pg/mL [33], whereas in typical adults, the levels typically remain below 7.8 pg/mL [34,35]. The severity of infection can be partially identified by the expression level of IL-6 according to earlier research [36]. However, while the results from the present study align with the majority of studies confirming the detrimental signaling promoted by elevated levels of IL-6, another study demonstrated that low levels of IL-6 are associated with an 8-fold higher risk of relapse in patients in treatment-free remission (TFR) of CML [37].

Similarly to what was described for IL-6, tumor necrosis factor (TNF) is another well-known pro-inflammatory cytokine that plays a role in various processes related to health and disease [31]. In accordance with the literature, not only are all leukocytes capable of producing TNF, but different tumor cells, including CML cells, also produce it, leading to increased systemic levels of TNF that are typically observed in patients with chronic myeloid leukemia [33]. In fact, TNF has been recognized as a key regulator at all stages of tumor malignancies, including tumorigenesis, cancer cell proliferation, survival, angiogenesis, cellular invasion, and metastasis [33]. Moreover, TNF can stimulate the release of other pro-inflammatory cytokines in larger quantities, thereby perpetuating chronic inflammatory responses [31,38,39]. Building upon this information, our study revealed that K-562 cells release elevated levels of TNF following stimulation by LPS. Similar to the results observed for IL-6, pretreatment with both concentrations of vitamin C (5 µg/mL or 10 µg/mL) substantially reduced TNF release, underscoring the potent capacity of vitamin C to suppress the hyperinflammatory response induced by LPS in K-562 cells. To further substantiate the ability of vitamin C to modulate the inflammatory response triggered by LPS in myeloid cancer cells, we also assessed the levels of anti-inflammatory cytokines in this study.

In this context, we assessed the levels of IL-10, a classical immunomodulatory and anti-inflammatory cytokine originally described as a cytokine synthesis inhibitory factor (CSIF), given its primary function of inhibiting cytokine production [40]. This cytokine is produced by various cell types, including dendritic cells, macrophages, B cells, different subsets of CD4+ and CD8+ T cells [33], and even lung structural cells [41]. IL-10 exerts its anti-inflammatory effects by inhibiting the activation and maturation of dendritic cells, as well as antigen presentation [42], resulting in the reduced release of many pro-inflammatory cytokines such as IL-1beta, IL-6, IFN-gamma, and TNF [40,41,42]. In our present study, we observed that LPS stimulation did not alter IL-10 production in K-562 cells. However, pretreatment with either 5 µg/mL or 10 µg/mL of vitamin C induced an increased release of IL-10 from these cells after LPS stimulation. This suggests a potential role for vitamin C in stimulating IL-10 release by myeloid cancer cells. In fact, low levels of IL-10 may pose a risk for transformation into leukemia [43]. In addition, favorable prognostic indicators for survival in patients with acute myeloid leukemia (AML) include reduced levels of IL-6 and elevated levels of IL-10 [44].

In addition to IL-10, another protein with anti-inflammatory, antioxidant, and anti-cancer properties involved in cancer biology is known as Klotho [45]. Initially characterized as an anti-aging protein, particularly in the context of kidney function [46], Klotho has since been associated with various age-related diseases, including cardiovascular, renal, musculoskeletal, and neurodegenerative conditions [47,48]. It is noteworthy that an increasing body of research has highlighted the potential role of Klotho in cancer biology [45], as both cancer and aging share underlying pathophysiological mechanisms, such as the time-dependent accumulation of DNA damage, genomic instability, increased mutagenesis, and reduced levels of Klotho [45,48,49]. Specifically, in our study, we observed that LPS stimulation led to a reduction in the release of Klotho by K-562 cells. Conversely, we also found that pretreatment with vitamin C at a concentration of 10 µg/mL was able to restore Klotho release by these cancer cells after LPS stimulation. In fact, other compounds, which also contains vitamin C in their composition, have been shown to increase Klotho release by K-562 cells stimulated with LPS [10], reinforcing the findings of the present study.

It is widely accepted that the modulation of pro- and anti-inflammatory responses can be attributed to various regulatory mechanisms [50,51,52]. In this context, purinergic signaling pathways, primarily mediated by the up-regulation and activation of the P2X7 receptor induced by increased extracellular ATP accumulation, have been identified as key mediators of multiple aspects of cancer progression [52,53]. Considering this knowledge, our study also aimed to investigate whether components of the purinergic signaling pathway (ATP accumulation and P2X7 receptor expression) could be involved in the hyperactivation of myeloid cancer cells induced by LPS. Our results demonstrated that LPS stimulation led to an increase in ATP release by K-562 cells, followed by an up-regulation of the P2X7 receptor in these cells. Furthermore, the present study observed that while pretreatment with vitamin C at both doses (5 µg/mL or 10 µg/mL) reduced ATP release, only the 10 µg/mL dose of vitamin C significantly inhibited the expression of the P2X7 receptor in K-562 cells following LPS stimulation.

According to the scientific literature, the P2X7 activation classically induces IL-6 release [54], whilst P2X7 deletion is capable of inhibiting this response [55]. Therefore, the remarkable effect of pretreatment of vitamin C in reducing both ATP release and P2X7 receptor expression means that not only can it putatively lead to a reduction in the production of pro-inflammatory cytokines, such as IL-6 and TNF, as found in the present study, but also allows us to suggest that components of the purinergic signaling pathway (ATP accumulation and P2X7 receptor expression) could be impacted by vitamin C in the context of LPS-induced hyperactivation of K-562 cells. In addition, a possible hypothesis to be further investigated is whether an autocrine pathway characterized by an ATP feedback loop stimulating P2X7 receptor expression via pannexin 1 (Panx1) channels may be involved in the response observed in the present study in K-562 cells, as previously demonstrated in other cell types, such as dendritic cells [56].

Supporting our hypothesis, a previous study demonstrated that vitamin C supplementation can restore the sensitivity of tumor cells to imatinib [57]. This led to a reduction in cancer cell proliferation, which, in part, can be attributed to its ability to down-regulate the production of pro-inflammatory cytokines like TNF in CML patients [18]. As a result, the hypothesis that vitamin C may serve as an adjunct therapy for chronic myeloid leukemia, enhancing the efficacy of imatinib which is one of the primary anti-cancer drugs, should be investigated in large-scale clinical trials. However, it is important to note that while our results emphasize the potential role of vitamin C in controlling or even inhibiting the hyperinflammatory process in chronic myeloid cancer cells (possibly through its modulatory effect on the purinergic signaling pathway strictly components, i.e., ATP accumulation and P2X7 receptor expression), our study is limited in that is only demonstrated these effects in the context of hyperactivation of myeloid cancer cells. In addition, a specific functional analysis of the P2X7 receptor in the observed effects needs to be further investigated. There is no evidence of these functional effects in other aspects of cancer biology, such as reducing cell proliferation, preventing leukemogenesis, or inducing malignancy in untransformed cells. Therefore, further studies are necessary to explore the potential of vitamin C in interfering in all stages of tumor malignancy processes.

Of note, autophagy has been proven to be a key pathway involved in CML [11,12,13,14,15]. Autophagy is a highly conserved cellular process responsible for maintaining cellular homeostasis by degrading and recycling damaged or dysfunctional cellular components [11,12,13,14,15]. It plays a critical role in various physiological and pathological conditions, including cancer [11,12,13,14,15]. In the context of chronic myeloid leukemia (CML), autophagy has garnered significant attention due to its complex and multifaceted relationship with the disease [11,12,13,14,15]. One of the key biomarkers associated with autophagy in CML is the microtubule-associated protein 1 light chain 3 (LC3). LC3, specifically LC3-II, is a widely used marker to monitor autophagosome formation, a crucial step in the autophagic process [12,58]. Studies have shown that autophagy can have a dual role in CML. On the one hand, it can promote cell survival and resistance to treatment, as CML cells may exploit autophagy to adapt to stress conditions, such as tyrosine kinase inhibitor therapy. On the other hand, excessive autophagy can also lead to autophagic cell death of CML cells [59]. In the present study, we found that vitamin C, notably the dose of 10 ug/mL, significantly reduced the expression of LC3-II, an effect followed by a reduction in the levels of pro-inflammatory cytokines, increase in the levels of anti-inflammatory cytokines, and down-regulation of purinergic signaling. However, we cannot prove a causal relationship between these effects and the purinergic or even autophagy signaling pathways, which must be addressed in future studies.

## 5. Conclusions

In conclusion, the present study demonstrated that vitamin C has the capacity to inhibit the LPS-induced pro-inflammatory status of chronic myelogenous leukemia cells, potentially through the inhibition of purinergic and autophagy signaling.

## Data Availability

All raw data will be freely available upon a reasonable request to the corresponding author. The data are not publicly available due to Brazilian law of data protection.
